# PTEN regulates EG5 to control spindle architecture and chromosome congression during mitosis

**DOI:** 10.1038/ncomms12355

**Published:** 2016-08-05

**Authors:** Jinxue He, Zhong Zhang, Meng Ouyang, Fan Yang, Hongbo Hao, Kristy L. Lamb, Jingyi Yang, Yuxin Yin, Wen H. Shen

**Affiliations:** 1 Department of Radiation Oncology, Weill Medical College of Cornell University, New York, New York 10065, USA

## Abstract

Architectural integrity of the mitotic spindle is required for efficient chromosome congression and accurate chromosome segregation to ensure mitotic fidelity. Tumour suppressor PTEN has multiple functions in maintaining genome stability. Here we report an essential role of PTEN in mitosis through regulation of the mitotic kinesin motor EG5 for proper spindle architecture and chromosome congression. PTEN depletion results in chromosome misalignment in metaphase, often leading to catastrophic mitotic failure. In addition, metaphase cells lacking PTEN exhibit defects of spindle geometry, manifested prominently by shorter spindles. PTEN is associated and co-localized with EG5 during mitosis. PTEN deficiency induces aberrant EG5 phosphorylation and abrogates EG5 recruitment to the mitotic spindle apparatus, leading to spindle disorganization. These data demonstrate the functional interplay between PTEN and EG5 in controlling mitotic spindle structure and chromosome behaviour during mitosis. We propose that PTEN functions to equilibrate mitotic phosphorylation for proper spindle formation and faithful genomic transmission.

## Abstract (web summary)

One of the cellular functions of the tumour suppressor PTEN is to maintain genome stability. Here, the authors show that PTEN depletion leads to mitotic spindle shortening and chromosome misalignment due to aberrant EG5 activation.

## Graphical abstract

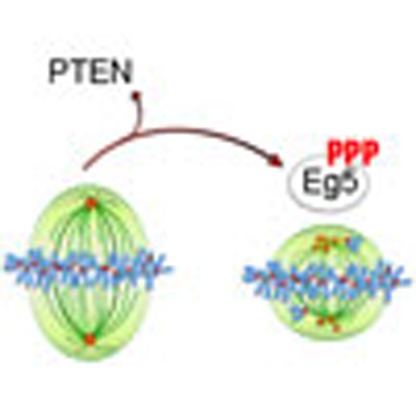

Chromosome instability is a hallmark of cancer and often stems from loss of tumour suppressor genes. *PTEN* is one such gene that is frequently mutated or inactivated in human cancers^1^,^2^. Loss of PTEN leads to structural and numerical chromosome aberrations^2^,^3^,^4^, suggesting that PTEN plays an important role in guarding the process of genetic transmission. We have previously shown that PTEN associates with centromeres to protect chromosomal integrity^4^. Our recent work pointed to the interplay of PTEN with histones in chromatin remodelling^5^,^6^ and revealed PTEN function in DNA replication^7^ and decatenation^8^. Mitosis is the central machinery for genetic transmission, during which chromosome congression advances segregation of each replicated pair of sister chromatids. Although PTEN has been implicated in mitotic regulation^4^,^9^, direct involvement of PTEN in the mitotic machinery remains largely unexplored.

PTEN was originally identified as a lipid phosphatase that antagonizes the PI3-kinase/Akt pathway^10^. Increasing evidence has revealed a number of protein targets of PTEN phosphatase such as focal adhesion kinase FAK^11^, transcriptional factors CREB1 (ref. 12) and IRF3 (ref. 13), and even PTEN itself^14^. These findings indicate the versatility of PTEN phosphatase in regulation of a variety of cellular processes.

Fidelity of chromosome segregation relies on the action of the mitotic spindle that uses dynamic microtubules plus multiple kinesin and dynein motors to generate forces required for mitotic chromosome movement^15^,^16^. EG5 is a member of the kinesin-5 family of plus-end-directed microtubule-based motor proteins^17^. Kinesin-5 motors display a conserved, bipolar homotetrameric organization consisting of two motor dimers lying at opposite ends of a central rod, allowing them to crosslink microtubules within the mitotic spindle and to coordinate their relative sliding during spindle assembly, maintenance and elongation^18^,^19^,^20^,^21^. EG5 is vertebrate kinesin-5 homologous to BimC in *Aspergillus nidulans*^22^,^23^, KLP61F in *Drosophila*^24^, Cut7 in fission yeast^25^, as well as Cin8 and Kip1 in budding yeast^26^. Phosphorylation of EG5 on an evolutional conserved site (Thr926 of human EG5, Thr925 of mouse EG5, Thr937 of *Xenopus* EG5, Thr1006 of BimC, Thr1011 of Cut7 and so on) in its C-terminal bimC box regulates its association with kinetochore microtubules as well as its function in spindle bipolarity and dynamics^24^,^27^,^28^,^29^,^30^. Current knowledge regarding functional relevance of EG5 phosphorylation status is mainly from studies in lower invertebrate species such as *Drosophila* and *Saccharomyces cerevisiae*, and reports on vertebrate species, particularly mammalian systems, are relatively limited. It is well known that EG5 is phosphorylated on Thr926 in the conserved bimC box by p34^cdc2^ kinase^27^. However, very little is known with regards to how phosphorylated EG5 can be dephosphorylated to achieve its functional balance.

In this report, we provide evidence that depletion of PTEN impairs chromosome congression leading to markedly increased incidences of unaligned chromosomes and resultant mitotic catastrophe, suggesting that PTEN plays an essential role in mitotic chromosome stability. In addition, PTEN is found to co-localize and interact with the critical mitotic motor EG5 during mitosis. We demonstrate that EG5 is a mitotic target of PTEN phosphatase and PTEN deficiency induces EG5 phosphorylation. Elevated phosphorylation levels of EG5 impair its association with microtubules and centrosomes, leading to spindle shortening and pole fragmentation. Phospho-dead EG5 can rescue these spindle defects in *Pten*-deficient cells. These data establish a functional link between PTEN phosphatase and EG5 motor protein in coordinating chromosome behaviour that is required for successful completion of mitosis and faithful chromosome transmission.

## Results

### Depletion of PTEN causes chromosome misalignment

**Figure 1:**
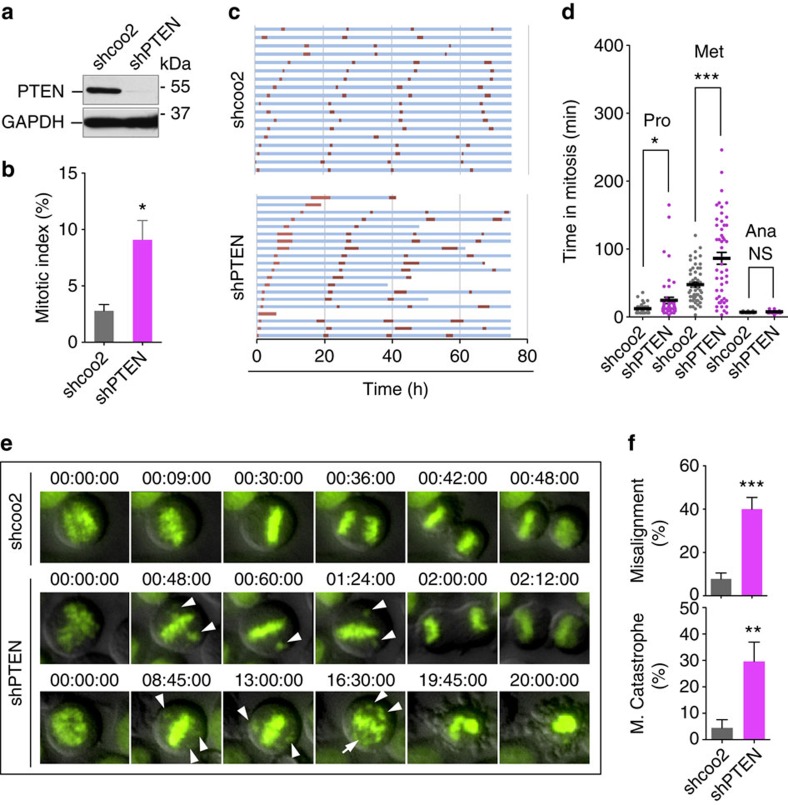
Depletion of PTEN leads to prolonged mitosis and defective chromosome congression. (**a**) Knockdown of PTEN. HeLa cells were transduced with a lentiviral shPTEN plasmid and PTEN expression was evaluated by western blotting and compared with cells containing the shcoo2 control vector. (**b**) Mitotic arrest by PTEN depletion. HeLa cells with and without PTEN knockdown were immunofluorescent stained with phospho-histone H3 (Ser10). Mitotic index was determined by flow cytometry as percentage of phospho-histone H3-positive cells. Data from three independent experiments were analysed by two-tailed *t*-test and presented as means±s.e.m. *P<0.05. (**c**) Prolonged mitosis in cells lacking PTEN. Time-lapse fluorescent microscopy was performed for 80 h for phase succession analysis in H2B-GFP cells with or without shPTEN. Interphase (blue) and mitosis (red) time lengths were scored in cells with and without shPTEN, showing 20 representative cells in each group. (**d**) Prominent metaphase delay upon PTEN knockdown. Mitotic timing was measured for different phases in cells of each group that completed mitosis during our analysis (*n*=100). Data are presented as means±s.e.m. and processed by one-way analysis of variance (ANOVA) and Newman–Keuls multiple comparison test. *P<0.05; ***P<0.001; NS not significant (*P*>0.05). (**e**) Comparison of mitotic progression in H2B-GFP cells with or without shPTEN shown by representative still frames of live cell microscopy. PTEN depletion results in frequent chromosome misalignment (middle panel) and subsequent mitotic catastrophe (bottom panel). Arrowheads point to chromosomes that fail to congress at the metaphase plate. (**f**) Summary of the frequency of chromosome misalignment (upper) and mitotic catastrophe (lower) in PTEN-depleted cells as compared to control cells (*n*>90). Data were analysed by two-tailed *t*-test. **P<0.01; ***P<0.001.

PTEN deficiency can disrupt chromosome integrity and result in aneuploidy^3^,^4^, which may attribute to erroneous chromosome transmission during mitosis^31^. To understand the importance of PTEN in chromosome inheritance during mitosis, we knocked down PTEN with two distinct shRNAs in HeLa cells (Fig. 1a; Supplementary Fig. 1a) and observed prominent mitotic arrest (Fig. 1b), which indicates an impeded process of chromosome transmission. To further document how PTEN regulates chromosome behaviour during cell division, we used time-lapse microscopy to monitor mitotic progression in cells expressing fluorescent histone H2B (H2B-GFP). An increased number of PTEN-depleted cells fail to survive the 80-h phase succession analysis, dying during or after a lengthy mitosis (Fig. 1c; Supplementary Fig. 1b). Examination of mitotic timing reveals that PTEN knockdown cells require a markedly longer time to complete chromosome congression, reflected by a prolonged metaphase (Fig. 1d). The majority of control cells congress their chromosomes on the metaphase plate within 50 min, and in contrast, over 20% of PTEN knockdown cells take longer than 200 min to achieve full alignment. Moreover, persistent congression failure often leads to mitotic catastrophe (Fig. 1e). As summarized in Fig. 1f and Supplementary Fig. 1c,d, PTEN knockdown results in a marked increase in the frequency of chromosome misalignment or catastrophic mitotic failure. These data suggest that PTEN is necessary for proper chromosome congression.

### Loss of PTEN impairs spindle architecture and pole integrity

**Figure 2:**
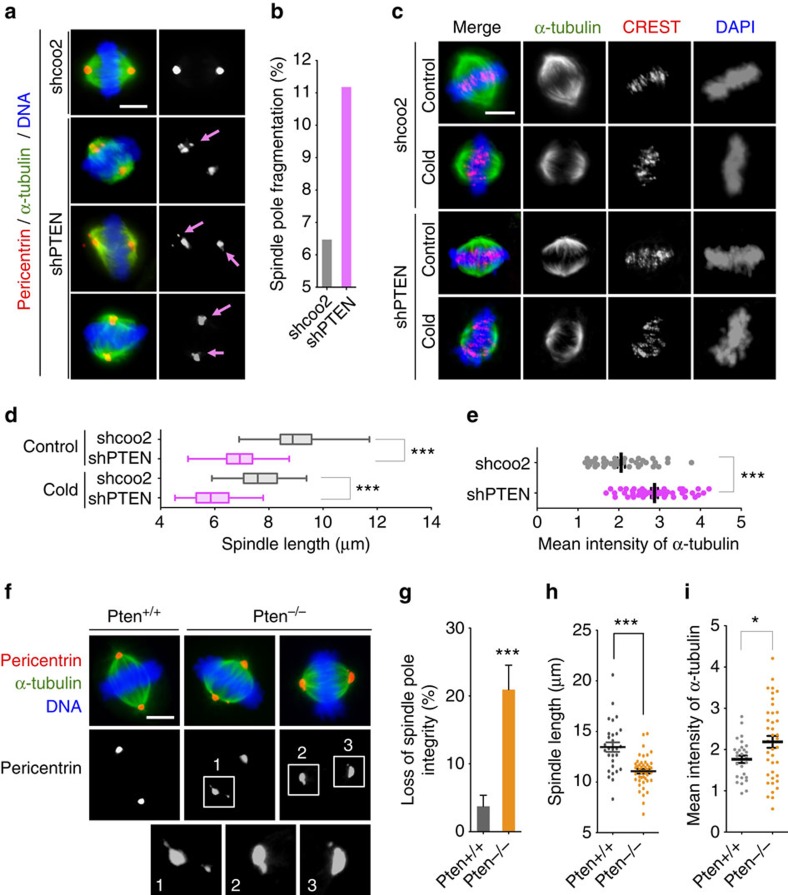
PTEN depletion impairs mitotic spindle geometry. (**a**) Spindle pole fragmentation in PTEN knockdown cells. PTEN knockdown and control cells were immunofluorescent stained for mitotic spindle (α-tubulin, green) and spindle poles (pericentrin, red). Arrows point to fragmented spindle poles. Scale bar, 5 μm. (**b**) Summary of the frequency of mitotic cells exhibiting spindle pole fragmentation in cells with and without shPTEN. (**c**) Shortening of mitotic spindles by PTEN knockdown. HeLa cells with and without shPTEN were left at 37 °C or placed at 4 °C for 10 min before immunofluorescence of microtubules (α-tubulin, green) and kinetochores (CREST, red) with DAPI counterstaining of chromosomes. Scale bar, 5 μm. (**d**) Box-and-Whisker plot showing the distribution of spindle lengths in PTEN knockdown cells and control cells with and without cold treatment. Data (*n*>50 in each condition) were analysed by one-way analysis of variance (ANOVA) followed by Bonferroni's multiple comparisons. ***P<0.001. (**e**) Summary of α-tubulin mean intensity within the spindle area in cells with and without PTEN knockdown. (**f**) Immunofluorescence of mitotic spindles (α-tubulin, green) and spindle poles (pericentrin, red) in *Pten*^*+/+*^ and *Pten*^*−/−*^ MEFs. Scale bar, 5 μm. (**g**–**i**) The frequency of spindle pole fragmentation (**g**), spindle pole distance (**h**) and α-tubulin mean intensity (**i**) is compared in *Pten*^*+/+*^ and *Pten*^*−/−*^ cells. Data are presented as means±s.e.m. and two-tailed *t*-test was used for data analysis. ***P<0.001.

Chromosome congression and subsequent segregation are mediated by the mitotic spindle, which is comprised of dynamic microtubules nucleated primarily at two centrosomes with intrinsic polarity^15^,^32^. It is thus plausible that disruption of chromosome alignment in PTEN-depleted cells may be due to the impairment of mitotic spindle organization. Indeed, multipolar and distorted spindles occur at a higher frequency and mitotic spindle poles in PTEN-depleted cells feature irregular shapes and display pole fragmentation, even when spindle bipolarity is properly established (Fig. 2a,b). These observations indicate that mitotic spindle pole integrity is compromised due to PTEN loss.

Spindle pole defects often cause kinetochore misattachment and kinetochore fibre (k-fibre) instability as geometrically defective spindles reduce the force generated on kinetochore microtubules and allow each kinetochore to interact with microtubules from more than one spindle pole^33^,^34^. To determine whether depletion of PTEN may impair spindle fibre stability, we investigated the morphology of spindle microtubules and their sensitivity to depolymerization at low temperatures. Much smaller metaphase spindles were observed in cells with shPTEN and further examination attributes the smaller-sized spindles observed in PTEN-depleted cells to a significantly reduced pole-to-pole distance (Fig. 2d; Supplementary Fig. 1e). However, the squatness of metaphase spindles in PTEN-depleted cells is unlikely due to k-fibre instability, as cold treatment reduces spindle length in cells both with and without shPTEN to a similar extent (Fig. 2d). To determine whether the reduction of spindle size in PTEN-depleted cells results in an overall lower quantity of tubulin component in metaphase spindles, we measured microtubule intensities. Surprisingly, PTEN knockdown cells display a significantly increased mean intensity of α-tubulin (Fig. 2e), indicative of microtubule condensation in a smaller spindle area.

To consolidate the geometric defects of the metaphase spindle observed in PTEN knockdown cells, we compared spindle morphology in *Pten*^*+/+*^ and *Pten*^*−/−*^ MEFs and found consistent spindle architectural abnormalities in *Pten* null cells. Cells lacking Pten display extra pericentric foci and fragmented spindle poles (Fig. 2f,g), and exhibit a significant reduction of spindle length (Fig. 2f,h) in metaphase as compared with wild-type cells. *Pten* deletion also results in condensed microtubules, likely due to smaller mitotic spindles (Fig. 2i). Altogether, these results illustrate that PTEN maintains normal spindle architecture to ensure faithful chromosome transmission during mitosis. As the control of spindle length represents a mitotic force-generating mechanism^35^, shorter spindle lengths in PTEN knockdown cells reflect a reduction of forces generated on chromosomes. Spindle pole fragmentation may also weaken centrosome-directed microtubule forces, leading to spindle shortening. Therefore, PTEN may sustain force generation through multiple mechanisms to promote proper spindle–chromosome interaction and efficient chromosome congression.

Previous studies showed that nuclear PTEN interacts with CENP-C to maintain centromere integrity, and PTEN also regulates the mitotic ubiquitin ligase complex APC/C in a phosphatase-independent manner^4^,^9^. We therefore tested the possible role of CENP-C and APC3 (an APC/C component) in mediating the mitotic function of PTEN. As shown in Supplementary Fig. 2a, metaphase spindle length remains unaffected by knockdown of either CENP-C or APC3, suggesting that PTEN maintains spindle size in a manner independent of its interaction with CENP-C or APC3. We next examined spindle pole integrity and found no effect of CENP-C depletion. In contrast, APC3 knockdown causes mitotic arrest and spindle pole fragmentation, whereas ectopic expression of APC3 can rescue spindle pole instability in *Pten*-deficient cells (Supplementary Fig. 2b,e). However, despite the significant alterations of spindle pole integrity caused by different APC3 status, the overall spindle morphology remains normal and stable following depletion of endogenous APC3 or ectopic APC3 expression (Supplementary Fig. 2a,f). These data suggest that spindle pole fragmentation in PTEN-deficient cells may not contribute to spindle shortening. Therefore, PTEN controls two independent mitotic functions, namely metaphase spindle length and spindle pole integrity, likely through distinct mechanisms. This notion was further confirmed by decoupling of the two phenotypes in a rescue experiment using a nuclear-excluded PTEN mutant (K13E;K289E)^36^. While PTEN^K13E;K289E^ retains the PTEN function in preventing spindle pole fragmentation, this mutant fails to restore spindle length in *Pten* null cells. More interestingly, it even exacerbates spindle shortening (Supplementary Fig. 2g,h). These data highlight the importance of PTEN nuclear localization to its mitotic function, which is predominantly reflected in the maintenance of mitotic spindle length.

### EG5 is a PTEN-associated mitotic protein

**Figure 3:**
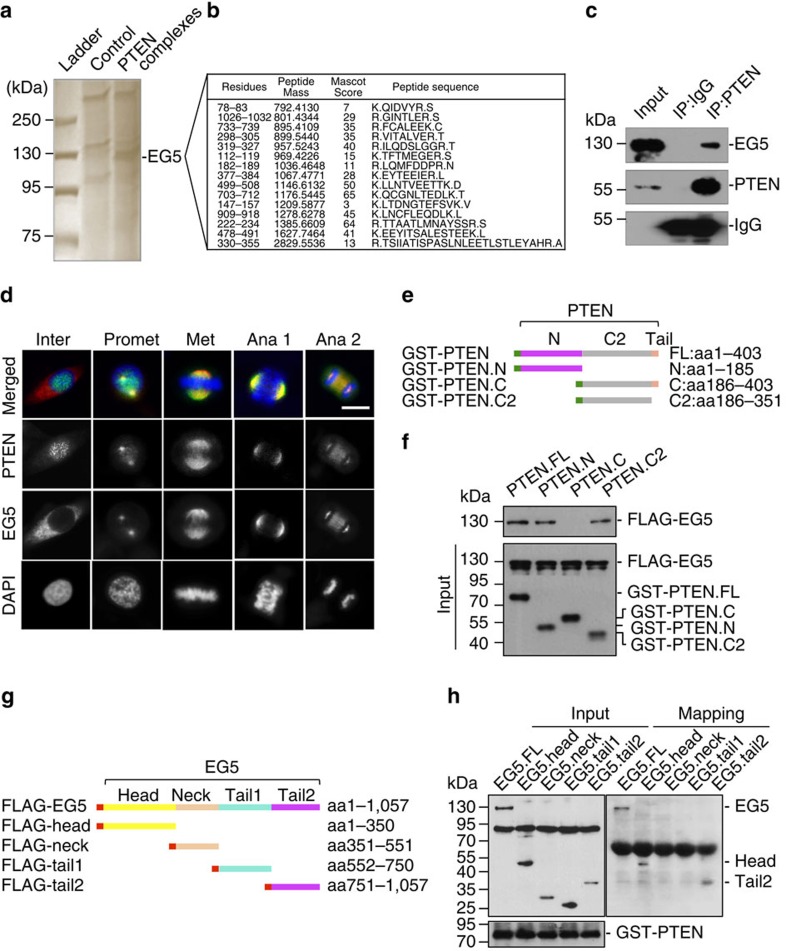
PTEN colocalizes and interacts with EG5 during mitosis. (**a**) Identification of EG5 as a component of PTEN-associated protein complexes by pull-down assay. HeLa cells containing FLAG-HA-tagged PTEN or a control vector were synchronized by releasing from double thymidine block for 8 h before tandem affinity purification and separation on SDS-PAGE gel. A 125-kD band was analysed by mass spectrometry (MS) revealing EG5 as a potential PTEN-associated protein. (**b**) A list of matched peptides from MS analysis. (**c**) Validation of the interaction between PTEN and EG5 *in vivo*. HeLa cell lysates were immunoprecipitated with an anti-PTEN monoclonal antibody followed by detection of EG5 by Western blotting. The same blot was probed with a polyclonal PTEN antibody. (**d**) Co-localization between PTEN and EG5 during mitosis. HeLa cells were fixed for immunofluorescent staining of PTEN (green in overlay images) and EG5 (red) before confocal microscopic analysis of spatial relationship between PTEN and EG5 during the cell cycle. DNA was counterstained with DAPI. Scale bar, 10 μm. (**e**) GST-tagged PTEN constructs for mapping the EG5-binding domains. (**f**) *In vitro* binding assay with FLAG-tagged full-length EG5 and GST-tagged different domains of PTEN as indicated. (**g**) FLAG-tagged full-length EG5 and different fragments were constructed for mapping the PTEN-binding region. (**h**) EG5 and its fragments with a FLAG tag as indicated in **g** were expressed in HEK293T cells (left panel) for *in vitro* interaction with Sf9-expressed His-PTEN. Ni-NTA purified protein complexes were subjected to FLAG and PTEN immunoblotting (right panel).

To gain further insight into the PTEN mitotic function, we sought to identify potential cellular factors in the PTEN pathway that control mitotic spindle architecture. Using an affinity purification approach, we identified EG5 as a potential PTEN-interacting protein (Fig. 3a,b; Supplementary Fig. 3a). EG5 plays a critical role in the dynamic assembly and function of the mitotic spindle by cross-linking and sliding adjacent microtubules^20^,^21^. In addition, EG5 function also contributes to spindle pole organization^18^. As a plus-end-directed microtubule motor, kinesin 5 mediates spindle–kinetochore interaction to control chromosome behaviour during congression and segregation^37^,^38^,^39^. To validate the pull-down results, we examined the interaction of endogenous PTEN with EG5 and detected PTEN-bound EG5 in both HeLa cells and *Pten*^*+/+*^ MEFs, but not in *Pten* null cells (Fig. 3c; Supplementary Fig. 3b). Further analysis using synchronized samples defines a mitosis-dependent interaction of PTEN with EG5 (detectable only during a period of 6–10 h after release from double thymidine block, Supplementary Fig. 3c). Moreover, an *in vitro* binding assay using purified recombinant proteins reveals a direct interaction between PTEN and EG5 (Supplementary Fig. 3d).

We next examined PTEN localization during mitosis to obtain an overall image of the spatial and temporal relationship between PTEN and EG5 (Fig. 3d). Interphase PTEN exhibits a diffuse staining in the cytoplasm but shows speckle-like foci in the nucleus. These nuclear foci denote kinetochores on chromosomes, as reported previously^4^. In contrast, EG5 is primarily localized in the cytoplasm during interphase. When cells enter prophase, the majority of PTEN is found on condensed chromosomes while some PTEN signals begin to appear around the centrosome region. Simultaneously, EG5 starts to accumulate on the centrosome and exhibit an overlap with PTEN. Starting from prometaphase, a very similar pattern of localization was observed for both PTEN and EG5. These molecules are enriched in the separating centrosomes at prometaphase, cover the whole spindle region during metaphase, accumulate at the spindle crest in anaphase 1, distribute to both the poles and the midbody in anaphase 2, and remain in the cleavage furrow by telophase (Fig. 3d). The only slight difference is that EG5 staining outlines the spindle microtubules, showing more detailed microtubule fibres, whereas PTEN only highlights the overall shape of the spindle, likely constituting the spindle matrix. These results clearly illustrate that PTEN is co-localized with EG5 during mitosis and further support their physical interaction, likely in a dynamic and mitosis-dependent manner. Further, domain mapping analysis showed that EG5 interacts with both the N-terminal phosphatase domain and the C2 domain of PTEN (Fig. 3e,f). Interestingly, we did not detect the interaction between EG5 and the tail-containing region of PTEN C terminus, which implies a possible interference by the PTEN C-terminal tail. Mapping of EG5 binding domains with PTEN revealed that PTEN interacts with either the N-terminal head domain or the C-terminal tail of EG5 (Fig. 3g,h).

### PTEN deficiency leads to increased phosphorylation of EG5

**Figure 4:**
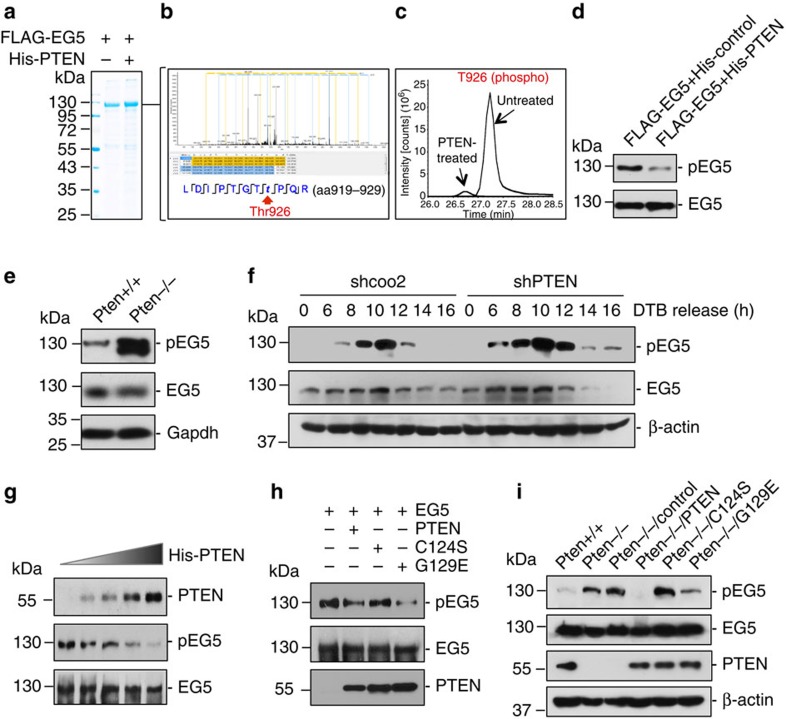
PTEN regulates EG5 phosphorylation. (**a**) PTEN treatment of EG5 for MS analysis of protein modification. Sf9-expressed FLAG-EG5 was incubated with or without His-tagged PTEN before FLAG immunopreciptation. The EG5 bands were excised from coommassie-stained gel for MS analysis of potential modification alterations. (**b**) A peptide of EG5 from the region aa919-aa929 identified by MS showing reduced phosphorylation at Thr926 in the PTEN-treated sample. (**c**) Quantification of phosphorylation at Thr926 in PTEN-treated and untreated samples. (**d**) Reduction of EG5 phosphorylation at Thr926 by PTEN. FLAG-EG5 treated with and without PTEN as prepared as in **a** was subjected to FLAG immunoprecipitation followed by immunoblotting analysis of EG5 phosphorylation using a site-specific (Thr926) phospho-EG5 antibody. (**e**) Elevation of EG5 phosphorylation at Thr926 in *Pten* null cells. *Pten*^+/+^ and *Pten*^*−/−*^ MEFs were analysed for EG5 phosphorylation and abundance by immunoblotting. (**f**) HeLa cells containing shPTEN or control shcoo2 were released from double thymidine block (DTB) for different periods of time followed by immunoblotting analysis of EG5 phospohrylation at Thr926. The same blot was probed with EG5 antibody to show EG5 expression levels. β-Actin was used as a loading control. (**g**) Dose-dependent reduction of EG5 phosphorylation by PTEN *in vitro*. Sf9-expressed His-PTEN protein was purified using a Ni-NTA agarose column. FLAG-EG5 expressed in Sf9 cells was immunoprecipitated with anti-FLAG M2 beads and incubated with increasing amounts of His-PTEN proteins, followed by western blot analysis of EG5 phosphorylation. The same blot was probed with EG5 antibody to show EG5 levels loaded in each lane. (**h**) Phosphatase-dependent reduction of EG5 phosphorylation by PTEN. His-PTEN proteins (including wild type and two mutants forms, C124S and G129E) were produced in Sf9 cells and purified using a Ni-NTA agarose column. FLAG-EG5 was incubated with equal amounts of His-PTEN proteins before examination of EG5 phosphorylation. Protein input of EG5 and His-PTEN (wild type and two mutants) is shown in the middle and lower panels from re-probing of the same blot with anti-EG5 antibody. (**i**) Reduction of EG5 phosphorylation by PTEN in phosphatase-dependent manner. *Pten*^*−/−*^ MEFs were transfected with vectors encoding FLAG-tagged wild-type PTEN or the phosphatase-deficient PTEN mutants PTEN^C124S^ and PTEN^G129E^, before immunoblotting assessment of EG5 phosphorylation at Thr926. The expression of EG5, PTEN (wild type as well as two phosphatase-deficient mutants) and β-actin was then evaluated by reblotting with corresponding antibodies.

EG5 function in binding mitotic spindles and mediating microtubule-kinetochore attachment is regulated by mitotic-specific phosphorylation^27^,^28^,^38^. To determine whether the mitotic-specific association of PTEN with EG5 may represent a functional regulatory relationship, we employed a mass spectrometric phosphoproteome approach to identify potential phosphorylation sites of EG5 that are regulated by PTEN (Fig. 4a). Incubation of EG5 with PTEN results in downregulation of multiple phosphorylation sites of EG5, among which Thr926 exhibits the highest basal level of phosphorylation indicative of physiological importance (Supplementary Table 1). Thr926 is an evolutionary conserved residue within the C-terminal tail domain that can be phosphorylated by Cdc2 (ref. 27). Mutations of this site impair normal EG5 localization and cause various spindle defects leading to erroneous chromosome segregation^27^,^29^,^30^,^38^. These studies demonstrate the importance of EG5 phosphorylation for its mitotic functions, although they have been carried out mostly in yeast or *Xenopus*. Evidence from mammalian systems is therefore necessary for understanding how EG5 phosphorylation is regulated and how EG5 deregulation affects spindle and chromosome behaviour in mitosis.

PTEN significantly reduces EG5 phosphorylation at Thr926 (Fig. 4b–d), suggesting PTEN may target this EG5 site for dephosphorylation. We therefore focused on EG5^Thr926^ for further investigation. We first examined *Pten* knockout MEFs and observed a higher level of EG5 phosphorylation but steady levels of total EG5 in *Pten* null cells as compared with wild-type cells (Fig. 4e), suggesting that PTEN targets EG5 to preferentially regulate its phosphorylation. We next analysed thymidine-synchronized HeLa cells with or without PTEN shRNA. Similar kinetics of mitotic-specific EG5 phosphorylation were observed with or without PTEN knockdown. When comparing each specific time point, we found a consistent elevation of EG5 phosphorylation in PTEN-depleted cells as compared with control cells (Fig. 4f). The expression levels of EG5 exhibit a slight increase during 6–8 h of DTB release independent of PTEN status. These data suggest that PTEN regulates EG5 activity by counterbalancing its phosphorylation.

### PTEN dephosphorylates EG5

To characterize EG5 as a mitotic target of PTEN phosphatase, we employed an *in vitro* phosphatase assay to determine whether PTEN could directly dephosphorylate EG5. Gradually increased amounts of PTEN result in a progressive reduction of EG5 phosphorylation at Thr926 without affecting the total levels of EG5 (Fig. 4g). To evaluate the importance of PTEN phosphatase activity in regulation of EG5 phosphorylation, we also examined the ability of two phosphatase-deficient PTEN mutants to dephosphorylate EG5. Incubation with PTEN^G129E^, which lacks lipid phosphatase activity, results in a significant reduction of EG5 phosphorylation, whereas the double-phosphatase-deficient His-PTEN^C124S^ mutant fails to do so (Fig. 4h). These results are consistent with our hypothesis that EG5 is a mitotic target of PTEN phosphatase and highlight the importance of the protein phosphatase activity of PTEN for dephosphorylation of EG5.

To further assess the role of PTEN phosphatase activity in suppression of EG5 phosphorylation *in vivo*, we examined the level of EG5 phosphorylation in *Pten* null cells with and without ectopic expression of wild-type PTEN or phosphatase-deficient PTEN mutants PTEN^C124S^ and PTEN^G129E^. EG5 phosphorylation is increased in *Pten* null cells and the introduction of wild-type PTEN significantly reduces EG5 phosphorylation (Fig. 4i). However, phosphorylation of EG5 remains at a high level in cells transfected with PTEN^C124S^, suggesting the requirement of PTEN protein phosphatase activity for dephosphorylating EG5. In contrast, the lipid phosphatase-deficient mutant PTEN^G129E^ retains the ability to suppress EG5 phosphorylation. Nevertheless, we noticed a remaining amount of EG5 phosphorylation in PTEN^G129E^-expressing cells, implying a possible involvement of the PI3-kinase/Akt pathway in regulating EG5 phosphorylation. To test this possibility, we treated both *Pten*^*+/+*^ and *Pten*^*−/−*^ cells with LY294002, a PI3-kinase inhibitor, before evaluation of EG5 phosphorylation. While LY294002 blocks Akt phosphorylation in the presence and absence of Pten, this inhibitor reduces phospho-EG5 only in *Pten*^*−/−*^ cells. In the presence of PTEN, a predominant EG5 regulator during mitosis, alterations of PI3-kinase activity no longer affect EG5 phosphorylation (Supplementary Fig. 4). Therefore, the suppression of EG5 phosphorylation by PTEN is unlikely dependent on its activity in antagonizing PI3-kinase.

### PTEN maintains EG5 interaction with the mitotic spindle

**Figure 5:**
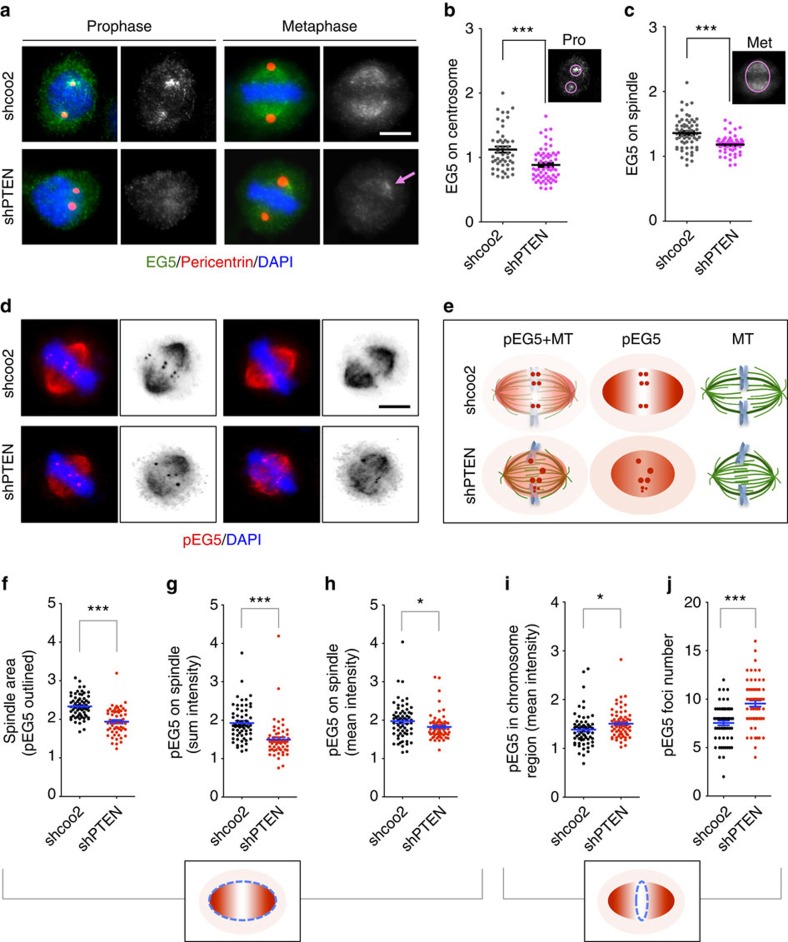
PTEN depletion causes aberrant distribution of EG5 on the mitotic spindle apparatus. (**a**) Co-immunofluorescence of EG5 (green) and pericentrin (red) showing reduced recruitment of EG5 to centrosomes (prophase) and spindles (metaphase) in PTEN knockdown cells. Gray scale of EG5 staining is also shown. Arrow points to aberrant EG5 enrichment in a region near the spindle pole. Scale bar, 5 μm. (**b**,**c**) Mean intensities of EG5 in the prophase centrosome region (**b**) or the metaphase spindle region (**c**) were measured as indicated in the upper-right corner of the scatter plots. (**d**,**e**) Immunofluorescence of phosphorylated form of EG5 (red). Scale bar, 5 μm. Note that PTEN knockdown cells lose a signal gradient along the spindle axis but exhibit an aberrant enrichment of phospho-EG5 in the spindle midzone, as depicted in the schemas (**e**). (**f**–**h**) Scatter dot plots summarizing the spindle area outlined by phospho-EG5 (**f**), sum intensity and mean intensity of phospho-EG5 in the spindle area (**g**,**h**). (**i**,**j**) Summary of phospho-EG5 mean intensity and foci number in the spindle midzone. Data are presented as means±s.e.m. and analysed by two-tailed *t*-test. *P<0.05; ***P<0.001.

The spatial and temporal dynamics of EG5 ensure its motor function in driving spindle formation and chromosome segregation. As PTEN regulates EG5 phosphorylation, we hypothesized that PTEN loss may affect the spatial relationship between EG5 and the mitotic spindle. Indeed, we observed aberrant EG5 distribution in PTEN knockdown cells. In prophase cells lacking PTEN, EG5 often fails to enrich at the centrosome region and the mean intensity of EG5 on centrosomes is significantly reduced (Fig. 5a,b). In the absence of PTEN, EG5 loses the spindle outlining property and the staining gradient from the spindle pole to the metaphase plate. With an overall reduction of the signal intensity on the metaphase spindle, EG5 often forms clusters near the spindle pole region (Fig. 5a,c). These observations suggest that PTEN is necessary for proper interaction between EG5 and the mitotic spindle apparatus.

Loss of PTEN results in elevation of the EG5 phosphorylation level, shown in the biochemistry assays (Fig. 4). To monitor and compare the localization changes of the Thr926 phosphorylated species of EG5 during mitosis, we performed phospho-EG5 immunofluorescence in wild type and PTEN knockdown cells. As a functionally active form of EG5 on the mitotic spindle, phospho-EG5 exhibits a similar overall distribution pattern in wild-type cells manifested by a clear spindle outline and a gradient along the spindle axis, leaving a prominent spindle midzone (Fig. 5d, upper panel). These discernable characteristics become obscure in PTEN knockdown cells (Fig. 5d, lower panel). As depicted in Fig. 5e and summarized in Fig. 5f,g,h, PTEN depletion results in a smaller spindle area, a reduction of phospho-EG5 intensity, and loss of signal gradient on the mitotic spindle. Relative to the enhanced microtubule density on the mitotic spindle (Fig. 2e,i) resulting from PTEN depletion, the loss of microtubule-associated EG5, both the total level (Fig. 5a–c) and the phosphorylated species (Fig. 5d–h), can be marked. We often noticed an increase of phospho-EG5 staining on the cell periphery around the mitotic spindle, implying that phosphorylated EG5 might be released from microtubules to spindle matrix or even beyond the spindle area. However, the intensity analysis did not show statistical significance, likely due to signal diffusion in a relatively larger cell peripheral space, particularly in PTEN knockdown cells with a smaller spindle area. Although PTEN depletion reduces the average signal of phospho-EG5 in the mitotic spindle, the phospho-EG5 staining in the chromosome region (spindle midzone) is significantly increased (Fig. 5i). This may reflect an increased interpolar microtubule overlapping in the spindle midzone (Fig. 5e), as PTEN depletion reduces spindle length without decreasing the quantity of spindle microtubules. Despite the similar overall distribution of EG5 and phospho-EG5 on the mitotic spindle, phospho-EG5 can form foci in the chromosome region of metaphase cells. These phospho-EG5 foci often appear in pairs although single dots and clusters are also common. It is unknown whether phospho-EG5 foci may be related to microtubule overlapping in the spindle midzone. Nevertheless, the phospho-EG5 foci number is significantly increased in PTEN knockdown cells as compared with wild-type cells (Fig. 5j), which at least partially contributes to the aberrant increase of phospho-EG5 staining in the chromosome region (Fig. 5i). In summary, depletion of PTEN significantly diminishes the spindle association of EG5, both the total level and the phospho-specific species, and alters their localization patterns.

### EG5 hyperphosphorylation mimics PTEN deficiency

**Figure 6:**
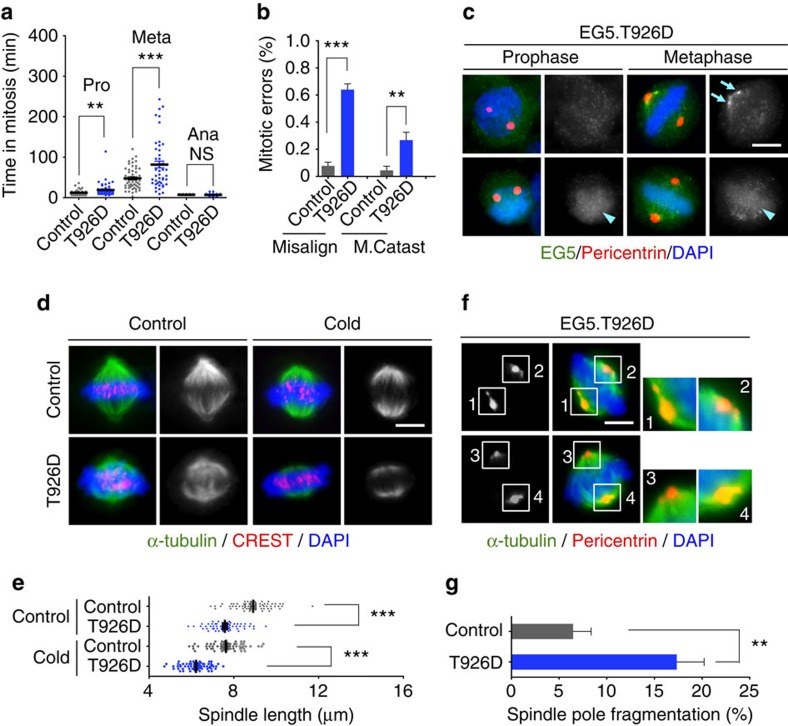
Phospho-mimetic EG5 imitates PTEN deficiency to impair chromosome congression and spindle geometry. (**a**) Phospho-mimetic EG5^T926D^ causes prolonged prometaphase and metaphase. Time-lapse microscopy was used to monitor mitotic progression in GFP-H2B cells transfected with the T926D mutant of EG5 or a control plasmid. Time durations of prometaphase, metaphase and anaphase were quantified in cells that completed mitosis (*n*>80). (**b**) Frequencies of different types of mitotic errors including chromososme misalignment and catastrophic mitotic failure were scored in cells with and without EG5^T926D^ (*n*>80 for each group). (**c**) HeLa cells with or without ectopic EG5^T926D^ were immunostained with EG5 (green) and pericentrin (red). Arrows point to regional aberrant enrichment of EG5 near the spindle pole and arrowheads indicate enhanced chromosome staining of EG5. Scale bar, 5 μm. (**d**) Shorter mitotic spindles in EG5^T926D^-expressing cells. Cells with and without ectopic EG5^T926D^ were cold-treated for 10 min before immunofluorescence of microtubules (α-tubulin, green) and kinetochores (CREST, red). Scale bar, 5 μm. (**e**) Scatter dot plots showing the distribution of spindle lengths in control cells and EG5^T926D^-expressing cells with and without cold treatment. Data (*n*>60 in each condition) were analysed with one-way analysis of variance (ANOVA) followed by Bonferroni's multiple comparisons. ***P<0.001. (**f**) Mitotic spindle pole fragmentation caused by EG5^T926D^, shown by immunofluorescence of metaphase spindles (α-tubulin, green) and spindle poles (pericentrin, red). Scale bar, 5 μm. (**g**) Quantification of spindle pole fragmentation (*n*=100) in the presence and absence of EG5^T926D^. Data are presented as means±s.e.m. and analysed by two-tailed *t*-test. **P<0.01.

It is known that EG5 phosphorylation is critical for association with microtubules for proper function^27^,^29^,^40^. However, it is unknown how uncontrolled hyperphosphorylation may impair EG5 function in spindle assembly and pole organization. In order to determine how aberrant EG5 phosphorylation affects mitosis, we constructed a mutant EG5 with the conserved site Thr926 substituted with aspartic acid to determine whether this phospho-mimetic mutant may mimic PTEN deficiency in causing mitotic errors. Live cell imaging analysis demonstrates that ectopic EG5^T926D^ causes mitotic delay or failure due to massive chromosome misalignment (Fig. 6a,b and Supplementary Fig. 5a,b), similar to what we observed in PTEN knockdown cells. Interestingly, cells containing ectopic EG5^T926D^ exhibit two distinct distribution patterns of EG5 in mitosis. In some cells, EG5 displays a diffuse staining without centrosome clusters in prophase or the spindle outline in metaphase. We also observed irregular aggregates of EG5 in the spindle pole region, as we saw in PTEN knockdown cells, and some foci overlap with spindle pole fragments (Fig. 6c). In other cells, EG5 exhibits a reduced staining on the spindle but an increased staining in the chromosome region. This phenomenon was observed in both prophase and metaphase (Fig. 6c). These distinct EG5 staining patterns may reflect varied expression levels of ectopic EG5^T926D^, in which a physiological level may imitate PTEN deficiency to impair normal EG5 distribution and an extremely high level may act dominant negatively leading to complete displacement of endogenous EG5. These data illustrate that EG5 hyperphosphorylation impedes its association with the mitotic spindle and leads to aberrant distribution and dynamics of this important motor protein.

To further determine how EG5 hyperphosphorylation influences spindle architecture and chromosome behaviour, we examined mitotic spindle morphology. Cells containing the EG5^T926D^ mutant exhibit shorter metaphase spindles and a markedly increased frequency of spindle pole fragmentation (Fig. 6d–g). To exclude the possibility that ectopic expression of EG5^T926D^ may produce a general kinesin-5 overexpression phenotype, we conducted the same experiments using HeLa cells transfected with wild-type EG5 (EG5^WT^). In contrast to spindle shortening induced by ectopic EG5^T926D^, overexpression of EG5^WT^ caused spindle elongation (Supplementary Fig. 5c). These distinct effects on spindle length may result from different status of EG5 on the metaphase spindle: EG5^T926D^ disrupts the normal EG5 distribution (Fig. 6c), whereas excessive EG5^WT^ may only increase its association with metaphase spindles without altering the distribution pattern. Further investigation is required to elucidate how EG5 hyperphosphorylation influences the formation of EG5 tetramers and resultant dynamics of the interplay between EG5 and mitotic spindles. Unlike the phosphomimeric EG5^T926D^ mutant, overexpression of EG5^WT^ does not alter the frequency of spindle pole fragmentation (Supplementary Fig. 5d). These results collectively indicate that spindle shortening and loss of pole integrity are EG5 hyperphosphorylation-specific phenotypes. These data also illustrate that deregulation of EG5 expression or phosphorylation often leads to distinct types of mitotic spindle disorganization. Therefore, proper levels of EG5 expression and phosphorylation are both essential for maintaining mitotic spindle morphology and function. Together with prolonged mitotic timing, chromosome alignment errors and mitotic catastrophe (Supplementary Fig. 4a,b), defective mitotic spindle architecture caused by EG5^T926D^ phenocopies PTEN deficiency or represents even more severe presentation of the same phenotypes. These data suggest that PTEN deficiency and EG5 hyperphosphorylation represent two facets of the same defective mitotic pathway that leads to aberrant spindle architecture and faulty chromosome behaviour.

### Spindle defects can be rescued by PTEN and phospho-dead EG5

**Figure 7:**
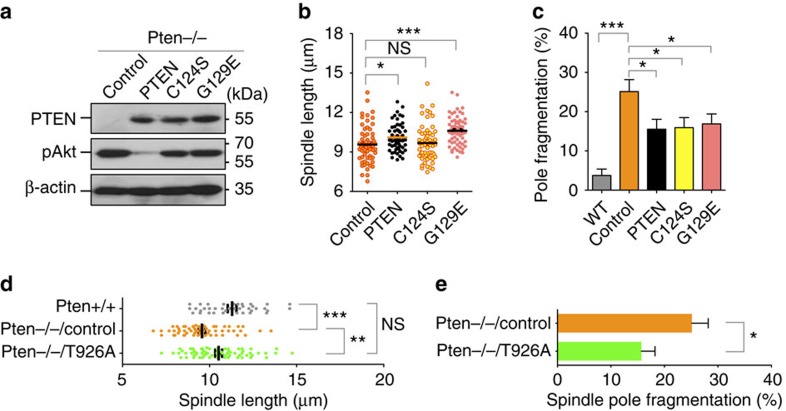
Shortened mitotic spindles in *Pten*-deficient cells can be rescued by both protein phosphatase-proficient PTEN and phospho-dead EG5. (**a**) Ectopic expression of wild type and phosphatase-deficient PTEN in *Pten* null cells. Akt phosphorylation was also shown to verify the lack of lipid phosphatase activity of PTEN mutants C124S and G129E. (**b**,**c**) The spindle length and pole integrity were analysed in *Pten*^*−/−*^ cells transfected with PTEN, PTEN^C124S^ or PTEN^G129E^ as indicated in **a**. Data are presented as means±s.e.m. and analysed by one-way analysis of variance (ANOVA) followed by Dunnett's multiple comparison tests. (**d**) *Pten*^*−/−*^ cells were transfected with EG5^T926A^, a phospho-dead mutant of EG5, or an empty vector (Control). Cells were subjected to immunofluorescent analysis of metaphase spindle pole distances. *Pten*^+/+^ cells were also included as a control. Data are presented as means±s.e.m. and analysed by one-way ANOVA followed by Turkey's multiple comparisons. (**e**) *Pten*^*−/−*^ cells with and without EG5^T926A^ were analysed for mitotic spindle pole fragmentation. Data were analysed by unpaired two-tailed *t* test. *P<0.05; **P<0.01; ***P<0.001.

To determine whether ectopic PTEN may rescue the defective spindle architecture and to evaluate the importance of PTEN protein phosphatase activity, we transfected *Pten*^*−/−*^ cells with wild-type PTEN as well as two PTEN mutants lacking the lipid or protein phosphatase activities (Fig. 7a). Wild-type PTEN significantly increases the spindle length and lacking of PTEN lipid phosphatase activity (PTEN^G129E^) does not abrogate this effect (Fig. 7b). In fact, the average length of mitotic spindles in cells containing the G129E mutant is even greater than that observed in cells with wild-type PTEN, which may be due to the difference of PTEN and G129E expression levels. In contrast, ectopic expression of a protein phosphatase-deficient PTEN mutant cannot rescue spindle shortening (Fig. 7b). These data demonstrate that PTEN relies on its protein phosphatase activity in maintaining mitotic spindle size. We also analysed spindle pole integrity and surprisingly found that all three forms of PTEN can partially reduce the frequency of spindle pole fragmentation to a comparable extent (Fig. 7c). These results, together with the data shown in Supplementary Fig. 2, suggest that spindle pole integrity serves as a convergent phenotype controlled by multiple distinct PTEN pathways, in a manner that may or may not require PTEN phosphatase activity or its nuclear function.

To further demonstrate the essential role of the PTEN-EG5 signalling axis in controlling mitotic spindle architecture, we tested our hypothesis that a phospho-dead EG5 mutant may be able to restore spindle length and pole integrity. We therefore constructed a non-phosphorylatable EG5 mutant by converting Thr926 to alanine. As shown in Fig. 7d, ectopic expression of EG5^T926A^ in *Pten*^*−/−*^ cells can increase spindle length to a comparable level as observed in wild-type cells. Moreover, EG5^T926A^ also reduces spindle pole fragmentation in *Pten* null cells (Fig. 7d,e). These data, together with those shown in Fig. 6, demonstrate the essential role of PTEN in maintain proper EG5 phosphorylation for spindle assembly and chromosome stability during mitosis.

## Discussion

PTEN has been recognized as a guardian of the genome since PTEN nuclear localization was linked to its function in chromosome protection and tumour suppression^1^,^4^,^36^,^41^,^42^. That loss of PTEN causes numerical and structural chromosome instability^2^,^3^,^4^,^7^,^8^,^43^,^44^,^45^ suggests PTEN may control the central process of genetic transmission in mitosis. In this study, we provide evidence to support a direct role of PTEN in the mitotic machinery by regulating EG5-mediated spindle assembly and chromosome congression at the kinetochore-microtubule interface. Our data also highlight the fact that the phosphatase activity of PTEN is required for tuning the balance of EG5 phosphorylation to prevent its deregulation and subsequent mitotic errors. The functional interplay of PTEN with EG5 constitutes an error-proof mechanism for protection of the genome by ensuring faithful chromosome transmission.

EG5 is regulated in a meticulous manner to ensure optimal levels of expression and phosphorylation during the cell cycle. Although EG5 can be phosphorylated by different upstream kinases including NIMA family protein kinase Nek6 (ref. 46) and aurora A^47^, EG5 phosphorylation by p34^cdc2^ on Thr926 is believed to be the primary modification event required for spindle localization and function^27^,^28^,^38^. Nevertheless, the effects of phosphorylation of this site differ in different systems. In vertebrates^28^ and *Drosophila*^48^,^49^, phosphorylation of this site enhances the localization of kinesin-5 to the spindle, whereas in *S. Pombe*^50^ phosphorylation of this site is dispensable for association with the mitotic spindle. While most of previous studies focused on the importance of EG5 phosphorylation, knowledge about whether aberrant phosphorylation of EG5 may function adversely and how EG5 is dephosphorylated for functional balance is limited. Our study demonstrates that PTEN phosphatase directly interacts with EG5 and inhibits EG5 phosphorylation. Our data suggest that EG5 phosphorylation must be precisely regulated as erroneous mitosis arises from either hypo- or hyper-phosphorylation of EG5. For example, hyperphosphorylation of EG5 at Thr926 in PTEN-deficient cells results in a reduced affinity to spindle microtubules and impairs the spindle architecture.

The reduced association of EG5, in particular the phosphorylated form, with the mitotic spindle shown by immunofluoresence in PTEN-deficient cells may seem to conflict with the increased level of EG5 phosphorylation shown in the biochemistry experiments. It is important to note that PTEN depletion results in mitotic arrest and the biochemistry experiments reflect a population behaviour of EG5 phosphorylation. Even at the single cell level, the deregulation of EG5 phosphorylation in PTEN-deficient cells is manifested mainly by alterations of spatial distribution patterns, including enrichment of phospho-EG5 in the chromosome region, accumulation of phospho-EG5 foci in the spindle midzone and release of phospho-EG5 from the spindle to the cell periphery. Our data suggest that PTEN controls a spatial and temporal coordination between EG5 and microtubules during mitosis through the maintenance of a proper equilibrium of EG5 phosphorylation.

PTEN depletion impairs mitotic spindle architecture, manifested by two interrelated yet independent forms of spindle disorganization, reduced spindle length and fragmented spindle poles. Although EG5 hyperphosphorylation can phenocopy both manifestations, spindle shortening appears to result exclusively from deregulation of the PTEN-EG5 pathway in a protein phosphatase-dependent manner. Defective spindle pole integrity, on the other hand, may rise from dysfunction of multiple PTEN pathways both dependent and independent of PTEN phosphatase activity. For example, phosphatase-independent regulation of the mitotic ubiquitin ligase complex APC/C may contribute to PTEN function in maintaining integrity of spindle poles. Interestingly, nuclear localization of PTEN may be dispensable for the maintenance of spindle pole integrity. However, PTEN requires its nucleus-importing activity to control the size of mitotic spindles. These observations suggest that PTEN controls multiple distinct aspects of mitotic spindle assembly and function. Both phosphatase-dependent and -independent mechanisms elaborate under the control of PTEN to ensure proper chromosome behaviour in the context of kinetochore-microtubule interaction.

Identification of EG5 as a mitotic target of PTEN phosphatase advances the current knowledge of PTEN function beyond its role as an antagonist of the PI3-kinase/Akt pathway. This work thus provides a reliable molecular marker of PTEN activity in mitosis. By targeting EG5 for dephosphorylation, PTEN claims its critical role in regulation of mitotic spindle-mediated chromosome congression and segregation. In combination with our earlier findings of PTEN association with centromeres (primarily during interphase) to protect chromosome integrity^4^, the current study reveals a new aspect of PTEN function in genomic stability through regulation of chromosome behaviour during mitosis. Altogether, PTEN not only constitutes the structural basis of chromosomes but also safeguards chromosome transmission.

## Methods

### Cell culture and antibodies

Primary mouse embryo fibroblasts (*Pten*^+/+^ and *Pten*^*−/−*^ MEFs) were prepared as described previously^45^. All other mammalian cell lines used in this study were from the American Type Culture Collection. Sf9 insect cells were from EMD Biosciences. Monoclonal anti-PTEN antibodies were purchased from Santa Cruz biotechnology and Cascade Bioscience. Anti-EG5 and anti-phospho-EG5 (Thr926) antibodies were from BioLegend (1:1,000 for western blotting and 1:200 for immunofluorescence).

### shRNA and plasmids

The pSUPER RNAi system (Oligoengine Inc.) was used to construct PTEN-specific shRNA expression plasmids and scrambled control shRNA. APC3 and CENP-C siRNAs were purchased from GE Pharmacon. The expression vectors containing FLAG or FLAG-HA tags were gifts from W. Gu^51^. The EG5 expression plasmid was created by ligating the full-length coding region of human EG5 into the expression vector with an N-terminal FLAG tag. The EG5 mutants, EG5^T926D^ and EG5^T926A^, as well as the nuclear-exclusive PTEN mutant, PTEN^K13E;K289E^, were generated using the QuikChange site-directed mutagenesis kit (Strategene) according to the manufacturer's protocol. Wild-type PTEN and phosphatase-deficient PTEN mutants, PTEN^C124S^ and PTEN^G129E^, have been described previously^4^. A pFastBac1 expression vector (Invitrogen) was used to construct plasmids expressing FLAG-tagged EG5, His-tagged wild-type as well as mutant PTEN proteins.

### Immunofluorescence and live cell imaging

Cells were fixed with 4% formaldehyde in PHEM (60 mM Pipes, 25 mM Hepes, 10 mM EGTA, and 2 mM MgCl2, pH 6.9) and permeablized for immunostaining. Spindle morphology was examined by staining microtubules and kinetochores with a monoclonal anti-α-tubulin antibody and CREST antisera, or a combination of α-tubulin and pericentrin. Images for co-localization of PTEN and EG5 were acquired using a Leica TCS SP5 confocal microscope. Dynamics of cell cycle progression through mitosis were analysed by time-lapse imaging using a Nikon Eclipse TiE microscope. Image acquisition was performed with a × 63 objective (PlanApo/1.4 numerical aperture) enclosed in a humidified incubation chamber with 5% CO_2_ at 37 °C and images were collected every 3 min for 30–80 h. Images of fixed cells were acquired as z stacks with 0.2–0.4 μm spacing to generate maximum-intensity projection of the entire cell. Imaging data were analysed using the NIS-Elements AR software.

### FLAG-HA pull-down and mass spectrometry

To purify PTEN-associated mitotic proteins for mass spectrometric analysis, HeLa cells stably expressing FLAG-HA-tagged PTEN and control cells containing an empty FLAG-HA vector were treated with nocodazole (100 nM) for 8 h. Nuclear fractions of cell extracts were subjected to sequential affinity purification using anti-FLAG M2 and anti-HA agarose beads and eluted with excessive FLAG and HA peptides. The final eluted materials were resolved by SDS–PAGE and a 125 kDa band in PTEN complexes was excised for mass spectrometric analysis.

### Phosphoproteomic analysis

On electrophoresis, the band was cut and gel digestion was performed according to standard procedures^52^. Phosphopeptides were enriched using chitosan-GMA-IDA-Fe(III) nanosphere^53^. Digests were re-dissolved and injected into an EASY-nLC 1,000 and analysed using 60 min elution program. Spectra were acquired on a LTQ Orbitrap Velos pro mass spectrometer. The Proteome Discoverer 1.3 software was used for data analysis dependent on IPI human v3.83 database. Label-Free Phosphopeptides Quantification was described elsewhere^54^ with minor changes. All the phosphopeptides contain the specific phosphorylated site were summed to represent the total level of site phosphorylation.

### *In vitro* protein–protein interaction

Recombinant proteins His-PTEN, FLAG-EG5, and a FLAG-tagged irrelevant protein, FLAG-Control, were expressed in asynchronous Sf9 cells for 72–96 h and purified with Ni-NTA beads (Qiagen) or anti-FLAG M2 beads (Sigma). Purified FLAG-EG5 (1 μg) or FLAG-Control (1 μg) was incubated with His-PTEN (1 μg) for 30 min at room temperature. Protein complexes were then immunoprecipitated with a PTEN monoclonal antibody and subjected to SDS–PAGE for detection of FLAG-EG5 using an anti-FLAG antibody.

### Dephosphorylation assay *in vitro* and *in vivo*

FLAG-EG5 protein was expressed in Sf9 cells and isolated by immunoprecipitation using anti-FLAG M2 beads. Sf9-expressed His-PTEN protein, as well as two mutant forms of PTEN, C124S and G129E, were purified and added to the FLAG immunoprecipitates and incubated at room temperature for 30–60 min. Samples were washed and subjected to western blot analysis of EG5 phosphorylation. For *in vivo* dephosphorylation assay, *Pten*^*−/−*^ MEFs were transfected with FLAG-PTEN, FLAG-PTEN^C124S^, FLAG-PTEN^G129E^ or an empty control vector. The level of EG5 phosphorylation was evaluated by immnoblotting with a site-specific anti-phospho-EG5 (Thr926) antibody.

### Data availability

All relevant data are available from the authors on request and/or are included with the manuscript.

## Additional information

**How to cite this article:** He, J. *et al*. PTEN regulates EG5 to control spindle architecture and chromosome congression during mitosis. *Nat. Commun.* 7:12355 doi: 10.1038/ncomms12355 (2016).

## Supplementary Material

Supplementary Information Supplementary Figures 1-6 and Supplementary Table 1
