# Supplementary material for: PTEN regulates EG5 to control spindle architecture and chromosome congression during mitosis
Source: Nat Commun. 2016 Aug 5;7:12355. doi: 10.1038/ncomms12355 (PMC4980451; doi:10.1038/ncomms12355)
Supplement: Supplementary Information — Supplementary Figures 1-6 and Supplementary Table 1 [file ncomms12355-s1.pdf]

Supplementary Figures

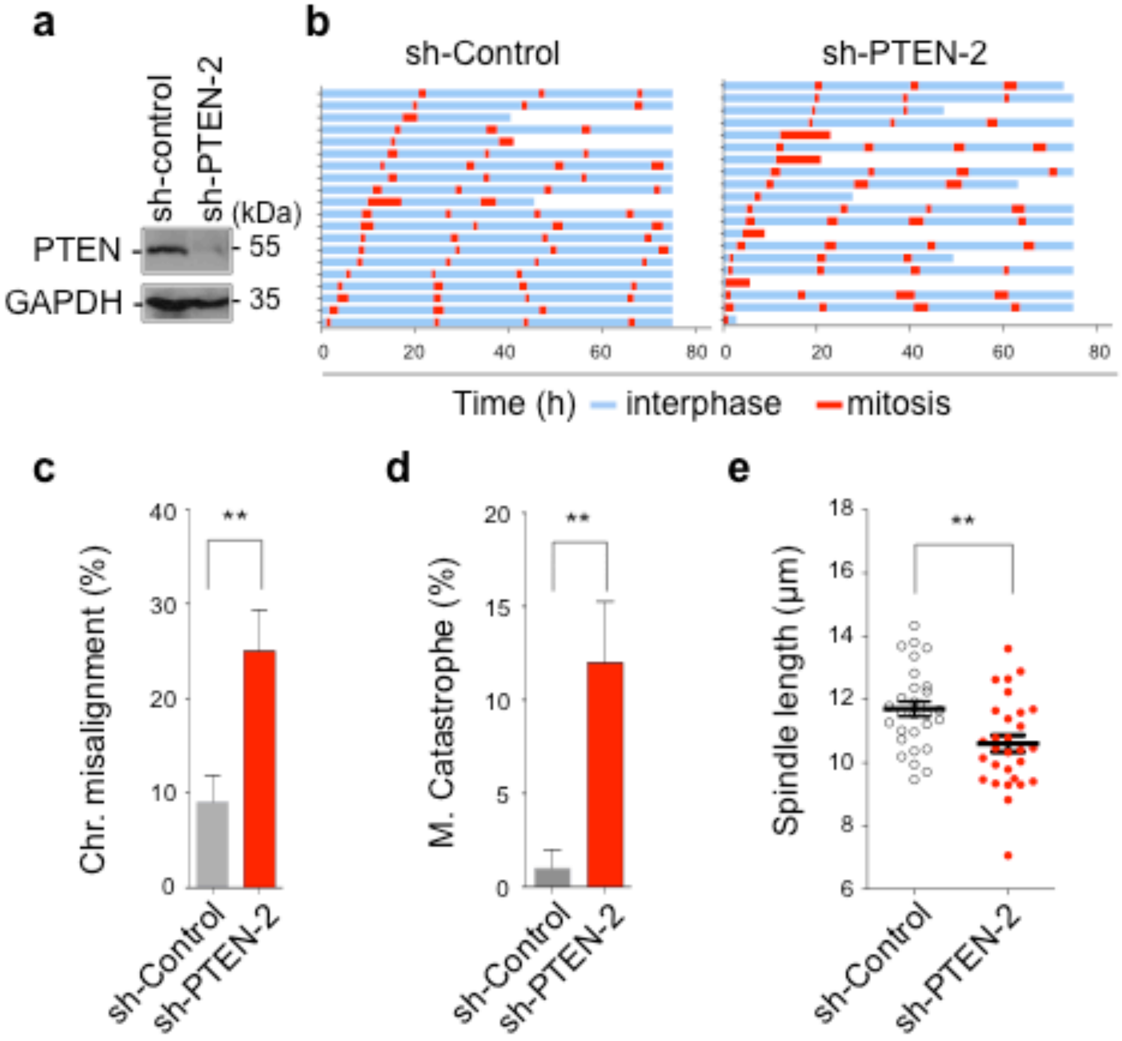

Supplementary Figure 1 | Verification of mitotic defects in PTEN knockdown cells using a short hairpin RNA (sh-PTEN-2) distinct from the one used in Figures 1 and 2. (**a**) Knockdown of PTEN in HeLa cells using sh-PTEN-2. (**b-d**) Cells with and without sh-PTEN-2 were transfected with H2B-GFP and subjected to live cell analysis of phase succession (b), chromosome congression defects (c) and mitotic catastrophe (d). (**e**) HeLa cells containing sh-PTEN-2 or sh-Control were analyzed for mitotic spindle lengths using immunofluorescence. Data are presented as means ± SEM and analyzed by two-tailed t-test. **, $p<0.01$.

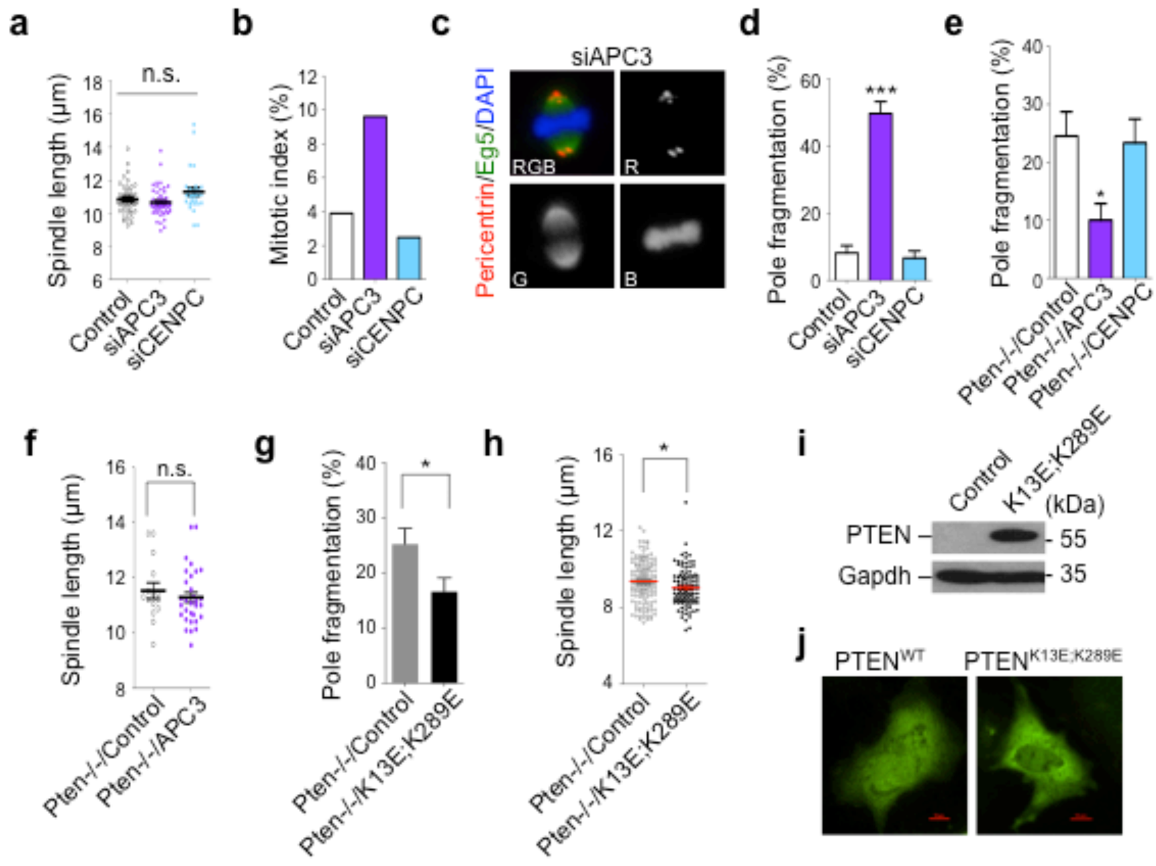

Supplementary Figure 2 | Role of APC3, CENPC and a nuclear-excluded PTEN mutant in maintaining mitotic spindle length and pole integrity. (**a-d**) HeLa cells transfected with siRNA for APC3 or CENPC were analyzed for spindle length (a), mitotic index (b) and pole fragmentation (d). A representative cells containing siAPC3 exhibit prominent spindle pole fragmentation (c). (**e-f**) *Pten*$^{-/-}$ MEFs were transfected with APC3 or CENPC expression plasmids and subjected to immunofluorescence analysis of mitotic spindle length and pole fragmentation. (**g-h**) Analysis of spindle architectural defects in *Pten*$^{-/-}$ MEFs containing a nuclear exclude PTEN mutant, K13E;K289E. (**i**) *Pten*$^{-/-}$ MEFs containing the K13E;K289E PTEN mutant and a control plasmid were used for immunoblotting analysis of PTEN expression. (**j**) *Pten* null cells transfected with wild-type PTEN and the K13E;K289E mutant were subjected to PTEN immunofluorescence to confirm nuclear exclusion of the K13E;K289E mutant. Data are presented as means ± SEM and analyzed by one-way ANOVA followed by Turkey's multiple comparison tests (b, d, e) or two-tailed t-test (f). *, $p<0.05$; ***, $p<0.001$.

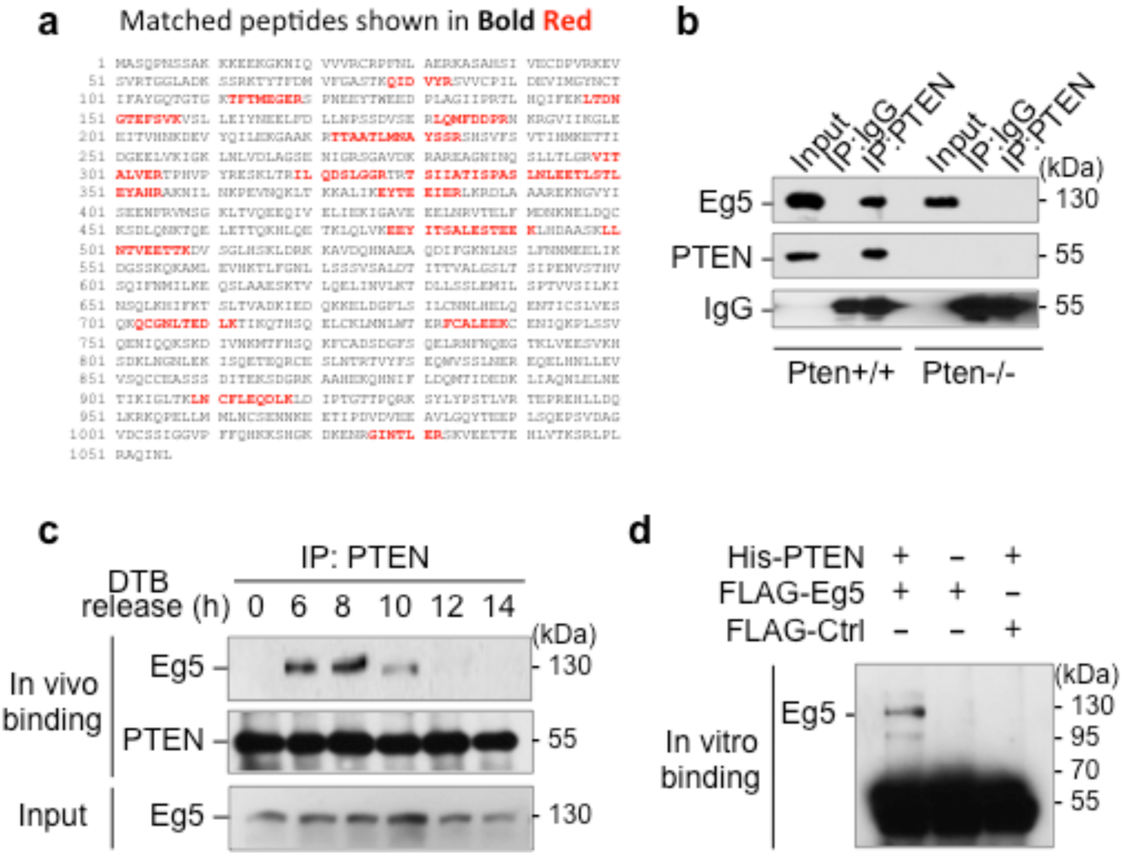

Supplementary Figure 3 | Physical association between PTEN and Eg5 during mitosis. (**a**) Eg5 protein sequence with matched peptides highlighted in bold red, revealed by MS analysis. (**b**) Coimmunoprecipitation of PTEN and Eg5 was performed in *Pten*$^{+/+}$ and *Pten*$^{-/-}$ MEFs, showing PTEN-bound Eg5 in wild-type cells but not *Pten* null cells. (**c**) Physical association of endogenous PTEN and Eg5 during mitosis. HeLa cells were synchronized by double thymidine block (DTB) and released for different periods of time. Cell lysates were immunoprecipitated by an anti-PTEN monoclonal antibody followed by detection of Eg5 by Western blotting. The same blot was probed with a polyclonal PTEN antibody to show equal PTEN levels in different immunoprecipitates. Eg5 input prior to immunoprecipitation is shown in the bottom panel. (**d**) Direct interaction of PTEN with Eg5 *in vitro*. Sf9 cells were infected with indicated recombinant baculovirus for 72 hours and subsequently harvested for protein purification using either an Ni-NTA agarose column or an anti-FLAG M2 affinity gel. Purified His-tagged PTEN (1 μg) was incubated with purified FLAG-Eg5 or a control protein (1 μg) followed by precipitation with anti-PTEN antibody. Samples were then subjected to extensive washes and subsequent analysis of FLAG-Eg5 expression in each interactive complex by Western blot analysis.

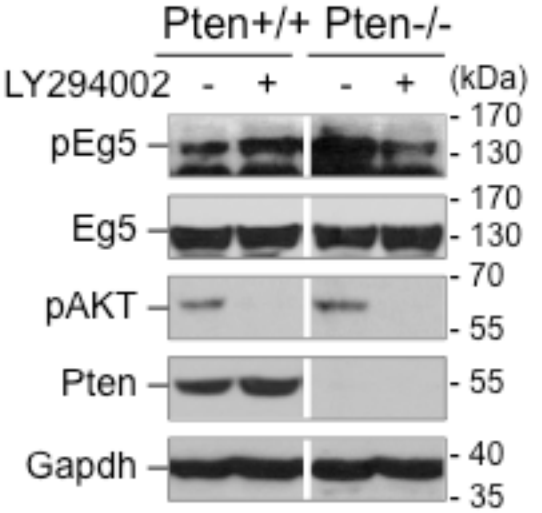

Supplementary Figure 4 | Effects of a PI3-kinase inhibitor on Eg5 phosphorylation. *Pten*$^{+/+}$ and *Pten*$^{-/-}$ MEFs were treated with LY294002 (20µM, 6h) followed by Western analysis of phospho-Eg5 (Thr926), Eg5, phospho-Akt (S473), Pten and Gapdh.

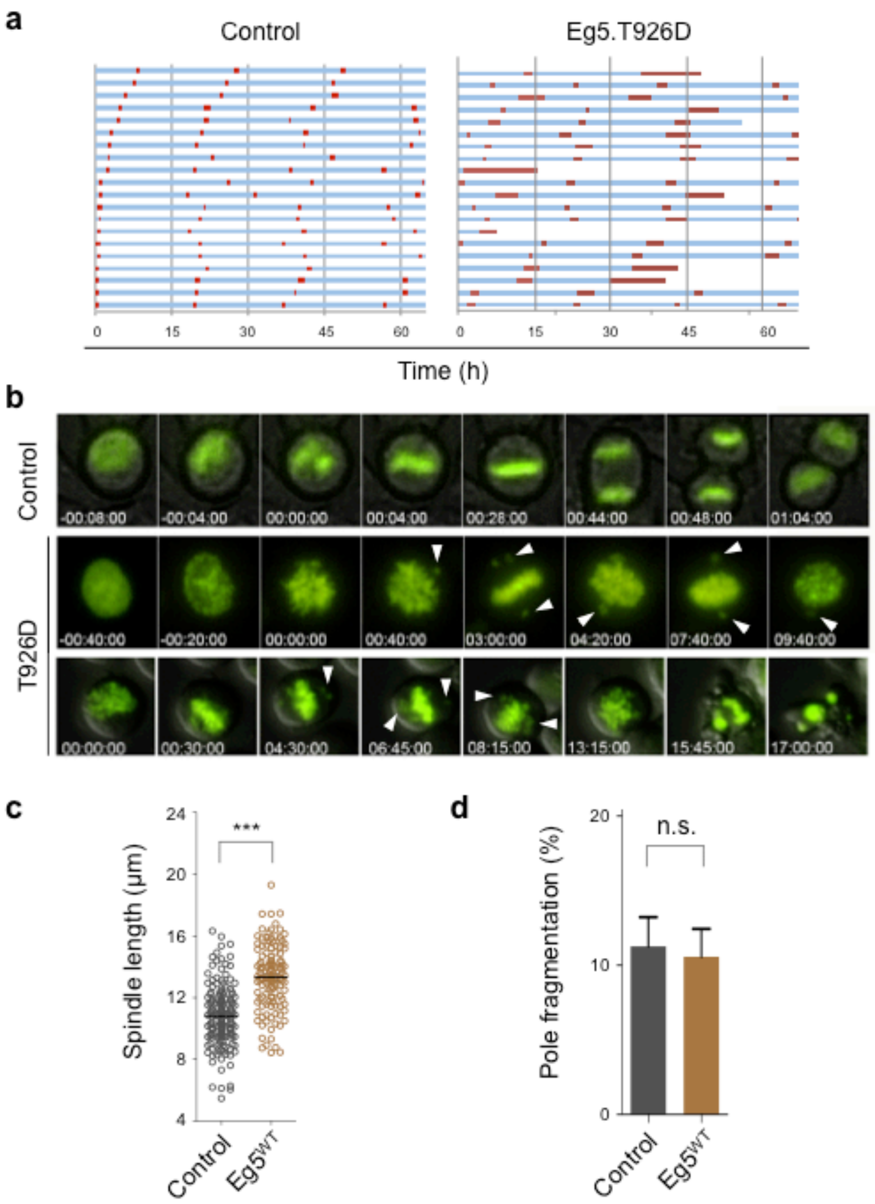

Supplementary Figure 5 | Hyperphosphorylation of Eg5 induces mitotic arrest, chromosome misalignment and mitotic catastrophe, whereas ectopic wild-type Eg5 causes spindle elongation. (**a**) Phase succession analysis of HeLa cells with and without overexpression of the $Eg5^{T926D}$ mutant. Blue, interphase; Red, mitosis. (**b**) Prolonged mitosis (middle panel) and mitotic catastrophe (bottom panel) by $Eg5^{T926D}$ due to chromosome misalignment, shown by time sequences of images from live cell microscopy. (**c-d**) HeLa cells were transfected with wild-type Eg5 prior to immunofluorescence analysis of mitotic spindle length and pole integrity. Data are presented as means ± SEM and analyzed by two-tailed t-test. ***, $p<0.001$; n.s., not significant.

Fig. 1a

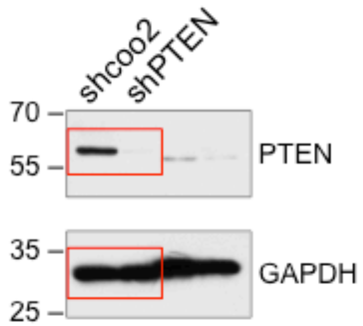

Fig. 3c

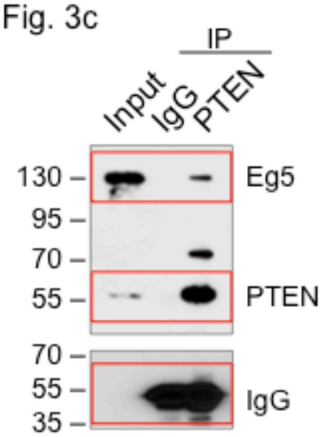

Fig. 3f

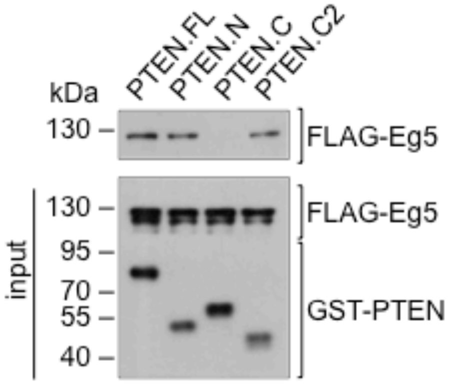

Fig. 3g

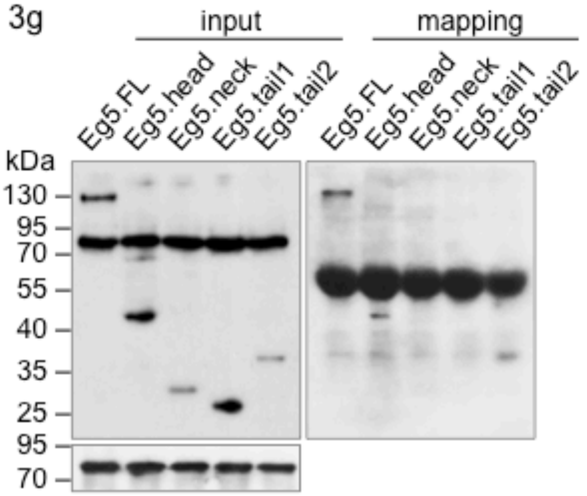

Fig. 4d

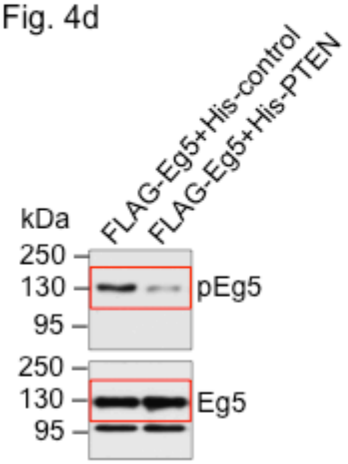

Fig. 4e

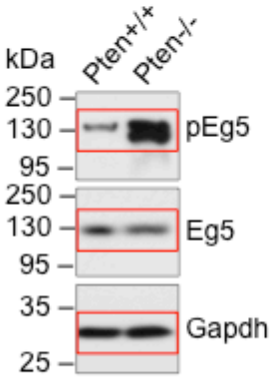

Fig. 4f

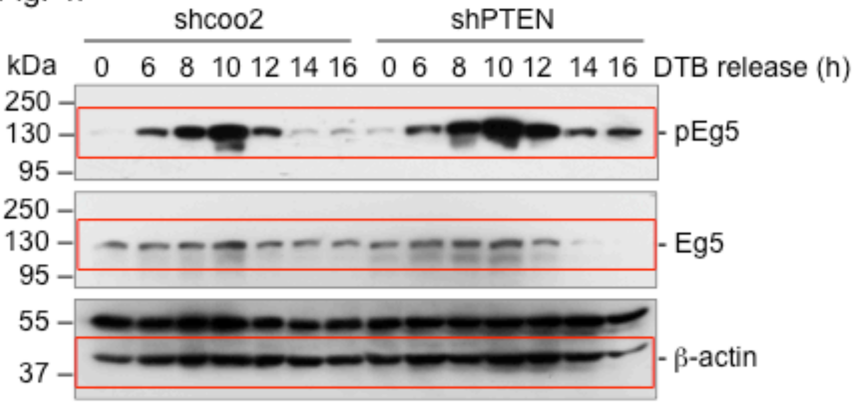

Fig. 4g

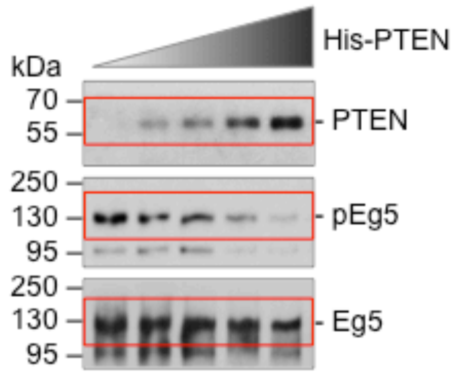

Fig. 4h

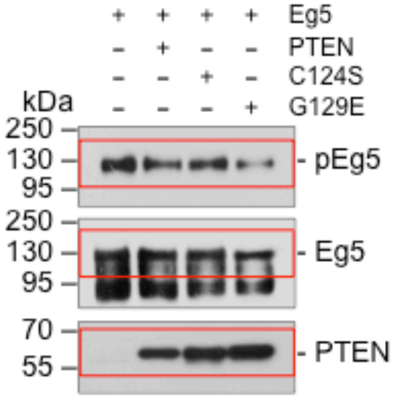

Fig. 4i

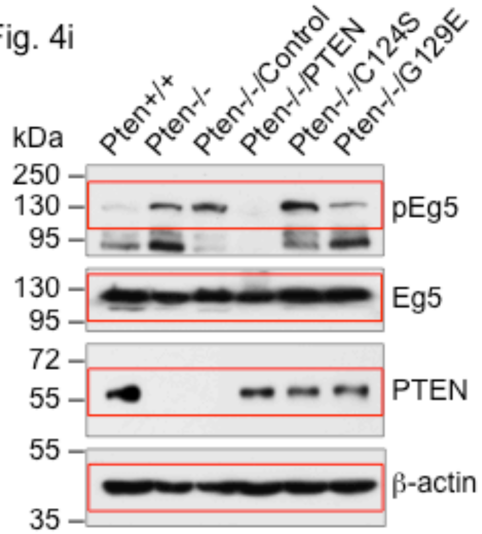

Fig. 7a

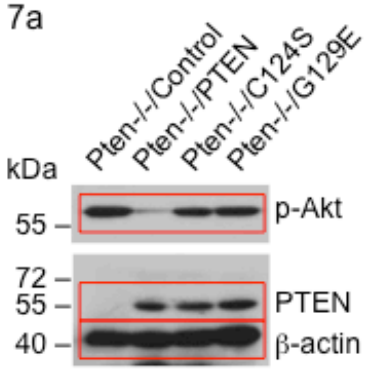

Fig. S1a

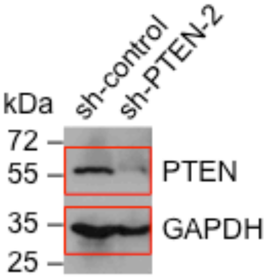

Fig. S2i

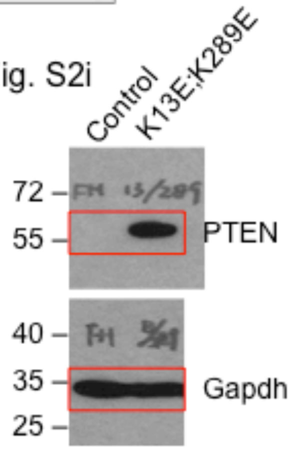

Fig. S3b

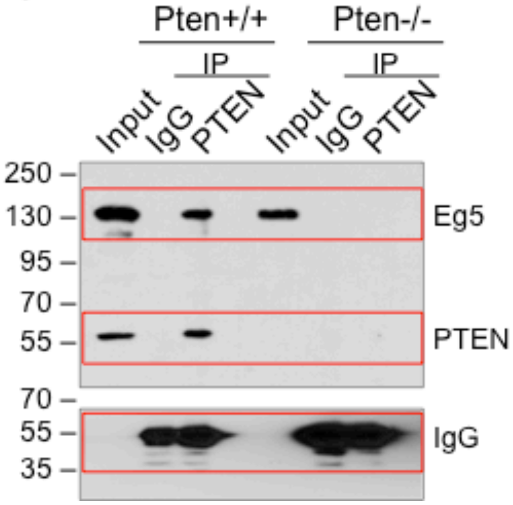

Fig. S3c

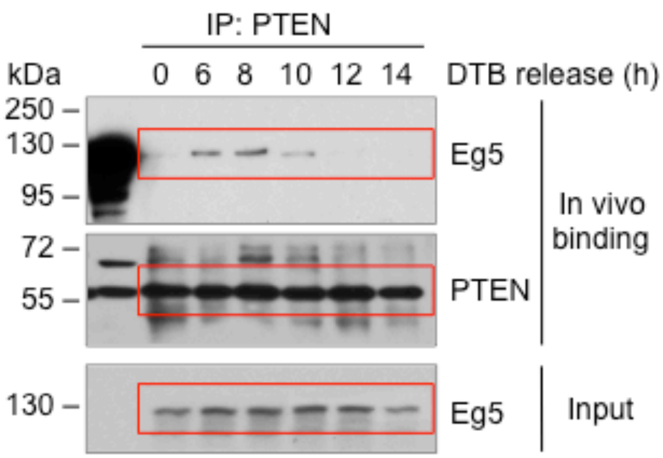

Fig. S4

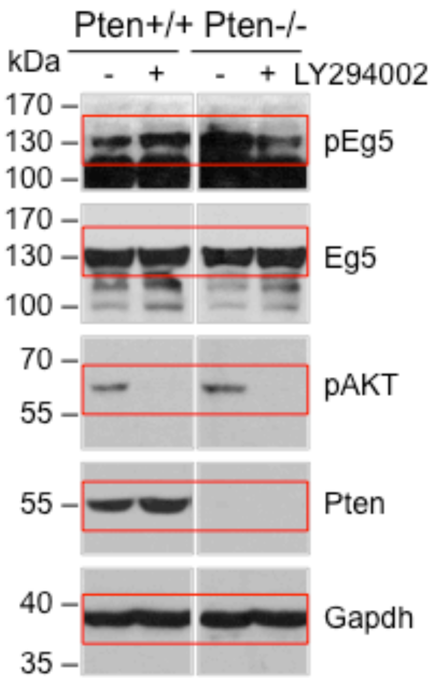

Fig. S3d

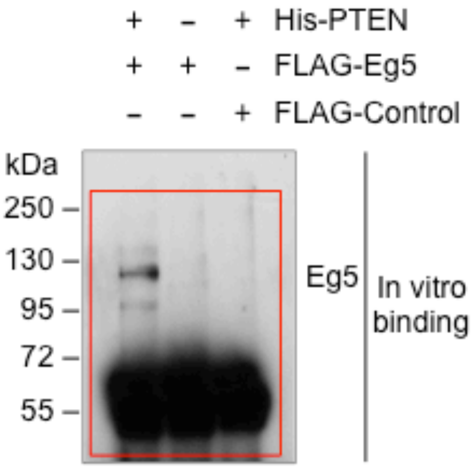

Supplementary Figure 6: images of uncropped blots

Supplementary Table

Supplementary Table 1 | Potential alterations of Eg5 phosphorylation sites revealed by MS analysis.

Intensity of p-peptides of EG5 ($x10^5$)

EG5	PTEN treatment	Control
S39	0.00	12.02
S233	0.00	16.36
S401	0.00	5.48
T458	0.00	1.74
S487	0.00	11.67
S748	0.00	5.56
S852	0.00	65.32
T926	1826.96	14674.92
